# Indoor exposure to particulate matter and volatile organic compounds in dwellings and workplaces and respiratory health in French farmers

**DOI:** 10.1186/s40248-019-0194-3

**Published:** 2019-10-02

**Authors:** Cara Nichole Maesano, Denis Caillaud, Hassani Youssouf, Soutrik Banerjee, Julie Prud’Homme, Christelle Audi, Kigninlman Horo, Yacouba Toloba, Ollivier Ramousse, Isabella Annesi-Maesano

**Affiliations:** 1 Epidemiology of Allergic and Respiratory diseases department (EPAR), Institut Pierre Louis d’Épidémiologie et de Santé Publique, INSERM, Sorbonne Université, Medical School St Antoine, 27 rue Chaligny, 75571 Paris CEDEX 12, France; 2 Service de Pneumologie, Hôpital Gabriel Montpied, CHU Clermont-Ferrand, Clermont-Ferrand, France; 3 Intensive Care Unit and Respiratory Diseases Department, CHU, Cocody, Abidjan, Côte d’Ivoire; 4 Service de pneumologie, CHU du Point-G, Bamako, E1631 Mali; 5 Mutuelle Salariés Agricoles, Clermont-Ferrand, France

**Keywords:** Asthma, Early airway obstruction, Chronic obstructive pulmonary disease, Air chemicals, Ultrafine particles, Total suspended particles, Rural, VOC, Multipollution, VOCs score

## Abstract

**Introduction:**

Few investigations have related objective assessments of indoor air pollutants to respiratory health in farmers, in spite of the many rural environmental hazards to which they are exposed. Chemical air pollution has been particularly neglected.

**Objective:**

We investigated the relationships of indoor exposure to particulate matter (PM) and volatile organic compounds (VOCs) to respiratory health in farmers.

**Methods:**

Nineteen VOCs (5 families) and PM (from ultrafine to total suspended particles (TSP)) were objectively assessed in dwellings and workplaces in 109 French farmers during a week. To take into account multiple exposures, scores of exposure were computed for total VOCs and VOCs families. Individuals filled a standardized questionnaire and underwent spirometry with bronchodilation test.

**Results:**

On average, VOCs concentrations were higher in dwellings than in workplaces. The reverse was observed for PM. When considering the mean concentrations of air pollutants for the whole farm (dwellings + workplaces), asthma (9.3%) was positively associated with elevated exposure to benzene (adjusted odds-ratio (ORa) = 6.64, 95%CI: 1.56–28.27), trichloroethylene (4.80, 1.00–23.30) and halogenated hydrocarbons score (2.9, 95% 1.3–6.8). Early airway obstruction (FEF_25–75_ < 80%, with normal FEV_1_ and FVC and FEV/FVC ≥ 70%) (29.8%) was related to elevated exposure to 2-butoxyetylacetate (11.49, 1.55–85.37) and glycol ethers score (2.0; 1.0–4.1) in the whole farm and to PM_2.5_ (ORa = 5.26, 95% CI: 1.09–25.28) in the granary/stable. The risk of Chronic Obstructive Pulmonary Diseases (FEV/FVC < 70%) (COPD) (4.26%) was found to be larger with elevated exposure to aldehydes (OR = 3.95, 1.09–14.26).

**Conclusion:**

Indoor chemical air pollution is detrimental to farmers’ respiratory health. More epidemiological investigations with detailed exposure assessments and clinical measures of respiratory effects are needed in rural settings to corroborate these findings.

## Introduction

Although the prevalence of active smoking is lower in farmers than in the general population, a high prevalence of chronic respiratory symptoms diagnosed as both chronic bronchitis and chronic obstructive pulmonary disease (COPD) has been found in famers and agricultural workers [1, 2]. Non-allergic asthma is also commonly found in farmers [3]. Furthermore, small airways diseases, pneumoconiosis and pathologic changes consistent with chronic bronchitis, emphysema and interstitial fibrosis predominate in farmworkers compared with non-farmworkers [3].

Respiratory disease in farm workers may be due to lifestyle-related and occupational characteristics of rural environments compared to urban environments. The rural work environment involves exposure to the physical hazards of weather, terrain, fires and machinery [4] as well as to various dust particles, including inorganic particles, such as silica, and various organic particles, such as molds, bacteria, endotoxins, mycotoxins, pollens, grains, and animal feed, found in different concentrations depending on the type of agricultural occupation [5–7]. In addition, farm workers may also be exposed to various gases during operations in confined animal feeding and to numerous chemicals used or generated on farms, such as pesticides, exhaust fumes, fertilizers, and nitrogen oxides [8–10]. New data have shown that due to the complexity of volatile organic compounds (VOCs), multi-pollution is an important issue to take into account when assessing the health effects of these air pollutants [11]. However, data on the health impact of exposure to indoor and outdoor air pollution in farms are scarce and these impacts deserve to be investigated.

The purpose of this study was to determine the effects on respiratory health from exposure to particulate matter (PM) of 4 sizes, Total Suspended Particles (TSP) and 19 VOCs (from 5 VOC families), objectively measured in dwellings and workplaces, among farmers drawn from the Environmental Factors of Allergic and Respiratory Diseases study (FERMA), conducted in the Auvergne region of France.

## Materials and methods

### Study population

Four hundred-ninety four (494) farmers from several cities in the Auvergne region were recruited between 2009 and 2010 during a compulsory occupational medicine visit conducted by the “Mutuelle des Salariés Agricoles” (MSA) in the context of the FERMA Study. Among them, 109 farmers living in 50 farms randomly selected were invited to participate in a follow-up including an air quality assessment of their home and workplace were randomly selected. Only a quarter of the initial population was targeted because of logistic and economic reasons. Indeed, the physician and the technician had to visit the farms there were spread in a large region. This survey was conducted from February to April 2012.

In our study, a farmer is defined as the owner of the farm and the farmed land or his/her spouse living in the farm. According to French rural code, a farm is defined as a workplace where agricultural activity is practiced, with a minimal area of installation defined according to the type of agricultural activity and managed by a farm operator.

### Air pollution measurements

Air pollution assessments were conducted in both the dwelling and the workplace by a team that visited each farm. More in detail, during the visit were measured 59 VOCs (living room, granary/stable) and the mass concentrations of different PM sizes and TSP (living room, bedroomgranary/stable,).

VOCs measurement was performed by radial diffusive with Radiello samplers® (https://www.sigmaaldrich.com/technical-documents/articles/analytical/radiello-air-sampler.html). Radiello samplers consist of a radial diffusive body made of porous polypropylene in which a cartridge with adsorbent is positioned. The adsorbent bed is selected for application and can consist of a pure adsorbent material or a chemically coated support. Due to this symmetry, analytes can access the adsorbent material throughout the 360° surrounding diffusive barrier/body resulting in a significant higher uptake rate. Two distinct Radiello samplers, numbers 145 and 165 respectively, were installed during a week in each setting. Sensors were placed at a height far from obvious sources of air pollution, such as a fireplace, stove, or newly purchased or cleaned furniture. Each farmer was instructed to uninstall the samplers and pack them in the envelope provided to send them for analysis at the end of the week. Radiello 165 cartridge absorbent with 2,4-dinitrophenylhydrazine *(*2,4-DNPH) coated Florisil® was used to assess aldehydes. Aldehyde-hydrazones formed in the cartridge were eluted by acetonitrile solvent and analyzed by liquid chromatography associated with a UV detector. Radiello 145 cartridge absorbent was used to assess BTEX (benzene, toluene, xylene) and other VOCs, which were extracted through thermodesorption and analyzed by gas phase chromatography equipped with flame ionization detection and/or mass spectrometry. Detailed methods are described elsewhere [12]. Nineteen VOCs among the fifty-nine measured were selected to study the potential impact of air pollutants on respiratory diseases based on their potential impact on air quality or comfort and their known toxicity, according to a hierarchical classification designed by a panel of experts (www.air-interieur.org). These Selected VOCs included three aldehydes (Acetaldehyde, Acrolein and Formaldehyde), twelve hydrocarbons (Benzene, 1,4-Dichlorobenzene, Etylbenzene, Styrene, Toluene, 1, 2,4-Trimethylbenzene, m/p-Xylene, o-Xylene, Tetrachloroethylene, Trichloroethylene, n-Decane and n-Undecane), and four glycols ethers (2-Butoxyethanol, 2-Butoxyetylacetate, 1-Metoxy-2-Propanol and 1-Metoxy-2-propylacetate).

PM ranging from 0.1 to 10 μm and Total Suspended Particulates (TSP) was assessed by a trained technician. A TSI P-track sensor (http://www.tsi.com/p-trak-ultrafine-particle-counter-8525/) in survey mode was used to display real-time particle number concentration (the number of particles present in a given volume) (pt/cm^3^) of ultrafine particle matter (UFPM) with diameters less than 0.1 μm (100 nm). An AEROCET 531S device (http://www.metone.com/particulate-aero531.php) was used in mass mode with a 2 min sample time to assess the mass concentrations of several sizes of PM (μg/m^3^) respectively. For the present study, 4 sizes of PM, namely UFPM, PM_1_, PM_2.5_, PM_10_ and TSP were taken into account based on their potential health impact, especially in urban areas [13, 14].

### Respiratory health outcomes

During the compulsory occupational medicine visit, a medical doctor assessed the weight, height, blood pressure, pulse rate, oxygen saturation of the farmers and also performed a lung function test using a MIR-Spirobank G spirometer system attached to a laptop computer with a reversibility test according to ERS / ATS guidelines. During the same visit, the individuals were interviewed by a nurse with a standardized questionnaire to assess health status and potential confounders, such as age, sex, tobacco smoking habit (classed as: never, ex or current smoking), educational level, presence of pets and molds at home, growing up on a farm, and wood heating.

Asthma was defined on the base of the following question: “Have you been diagnosed with asthma by a physician?” and confirmed through the medical records. COPD and early airway obstruction were defined using spirometry. Both were assessed after bronchodilation (with Salbutamol) during a reversibility test. COPD was defined according to the GOLD criteria, as forced expiratory volume at 1 s [FEV_1_], and forced vital capacity [FVC], ratio namely FEV_1_/ FVC < 70% [15]. In addition, a reduction in forced expiratory flow at 25–75% of the pulmonary volume, namely FEF_25–75_ < 80%, with normal FEV_1_ and FVC and FEV / FVC ≥ 70% after a detailed analysis of the volume curve by one of the authors (JFB) was considered as a proxy of early airway obstruction.

We present in this paper only the data of the farmers having had air pollution assessments.

### Statistical analyses

Descriptive analyses were performed using proportions in the case of qualitative variables and means with standard deviation (SD), minimum, maximum and interquartile ranges of the distributions in the case of quantitative variables, such as the concentrations of air pollutants as well as the number in the case of UFPM. Air pollutant distributions were available in living room, bedroom and granary/stable for PM and living room, granary/stable for VOCs. Correlations between quantitative variables were checked with Spearman’s rank correlation coefficient.

To avoid the linearity problem in the relation between the air pollutant and the health outcome, a binary exposure variable (elevated vs. low) was introduced. Exposure was considered as elevated if the concentration of air pollutant in the whole farm was higher than the 3rd quartile value. In contrast, if the concentration of air pollutant was lower than the 3rd quartile value, the exposure was considered as low.

To take into account correlations among VOCs, a total VOCs score variable and specific VOC score variables were created, according to the VOC family as previously described [10]. For each setting, each individual VOC concentration was categorized as 0 if less than the 3rd quartile value and 1 if greater. The total VOCs score was then defined as the sum of the categorized VOC concentrations and ranged from 0 to 19. In practice, the total VOCs score represents the number of VOCs in the farm for which elevated (>3rd quartile value) concentrations were found. Additionally, 5 specific VOC scores, one for each VOC family, were built using a similar approach, by summing categorized VOC concentrations by family: 1) aromatic hydrocarbons (benzene, toluene, m/p-xylene, O-xylene, 1,2,4-trimethylbenzene, ethylbenzene and styrene), 2) aliphatic hydrocarbons (n-decane, n-undecane), 3) halogenated hydrocarbons (trichloroethylene, tetrachloroethylene, 1,4-dichlorobenzene), 4) glycol ethers (1-methoxy-2-propanol, 2-butoxyethanol, 1-methoxy-2-propylacetate and 2-butoxyethylacetate) and 5) aldehydes (formaldehyde, acrolein and acetaldehyde).

Relationships between exposure to air pollutants and health outcomes were analyzed using the generalized estimating equation approach (GEE) with an exchangeable covariance matrix to adjust for correlations within people belonging to the same dwelling through the GENMOD SAS procedure, while taking into account potential confounding factors. In the case of total and specific VOC scores, the scores were fitted as continuous variables. The GEE model characterizes the marginal expectation (average response for observations sharing the same covariates) as a function of covariates. This approach was needed because individuals within the same farm tend to be more alike in terms of attitudes and behaviors, their environment and, for some of them, genetics than individuals from different farms. For consistency, all models were adjusted for the same confounders. This decision was also justified by the fact that health outcomes (asthma, COPD and early airway obstruction) share many risk factors. Bivariate marginal analysis between potential confounders and both outcomes were performed. All variables associated with asthma, COPD or early airway obstruction with a *p* < 0.30 were considered as potential confounders. Selected covariates that were collinear to others were omitted. Age, sex and smoking status were included in all models regardless of whether they changed the effect estimates significantly because they have been included as potential confounders in comparable studies [9, 10, 16].

The sample size of 109 subjects showed a statistical power of 80% and a 95% confidence level to evaluate associations between exposure to air pollutants and health outcomes with an OR of 2 or more.

## Results

### Description of population and prevalence of respiratory symptoms

Measurements of air pollution were conducted in 50 farms located in three cities of the Auvergne region: Riom-ès-montagnes (19 farms), Saint-Sauve (19 farms) and Saugues (12 farms). One-hundred and nine eligible farmers completed the health questionnaire. Fifty nine percent (59.3%) of respondents were male and 40.7% female. Farmers’ origin was distributed as following: 33% from Riom-ès-montagnes, 40.4% from Saint-Sauve and 26.6% from Saugues. As presented in Table 1, 38.0% of farmers were between 15 and 45 years old, 53.7% were between 45 and 65 years old and only 8.3% were older than 65 years. Seven percent (7.3%) of farmers were current smokers, and 29.4% were former smokers. Thirty-two percent (32.1%) had completed high school, 62.0% had grown up on a farm and 89.8% had worked in agricultural settings before. Concerning the farm characteristics, 77.1% of farmers declared they have pets, 5.5% declared the presence of mold and fungi in the house while water infiltration and humidity in the dwelling was declared by 14.1 and 29.4% of farmers, respectively. Demographics and farming characteristics according to the questionnaire in farmers participating in the survey investigating air pollution and those of the initial survey were similar (data not shown).

**Table 1:** Socio-demographical characteristics among farmer participants

	n	(%)
Population characteristics (*n* = 109)		
Sex (male)	64	(59.3)
Age (years)		
[15–45]	41	(38.0)
[45–65]	58	(53.7)
> =65	9	(8.3)
Smoking status		
*Curent smokers*	8	(7.3)
*Former smokers*	32	(29.4)
*Non-smokers*	69	(63.3)
*Education level*		
*< =High school*	74	(67.9)
*> High school*	35	(32.1)
Lived on a farm as a child	44	(62.0)
Worked in agricultural area before	97	(89.8)
Farm characteristics		
Presence of pets	84	(77.1)
Presence of mold, fungi	6	(5.5)
Water infiltration in the dwelling last 12 months	13	(14.1)
Humidity in the dwelling in the last 12 months	27	(29.4)
Persistent condensation	21	(19.6)
Farm near a heavy traffic area	23	(25.6)
Month of visit		
February	32	(29.4)
March	35	(32.1)
April	42	(38.5)

Table 2 presents the distribution of respiratory symptoms and diseases as reported by the farmers. In the past 12 months, shortness of breath after intense effort (18.4%) was the most prevalent symptom, followed by short breath (16.8%), morning cough (16.5%), and phlegm (13%). Forty-eight farmers (44%) declared having respiratory symptoms, such as sneezing, coughing, wheezing, shortness of breath, or dyspnea when handling reaped plants. In terms of diseases, 9.3% of farmers declared an asthma diagnosis by a physician. 29.8% presented early airway obstruction and 4.3% and COPD as defined with spirometry. Spirometry values did not differ significantly between asthmatics and non-asthmatics (not shown in the table).

**Table 2:** Frequency of respiratory symptoms and diseases among farmer participants

Symptoms	Total		Male		Female	
	N (%)		N (%)		N (%)	
Morning cough in the past 12 months	18	(16.5)	15	23,44%	3	6,67%
Phlegm in the past 12 months	14	(13.0)	11	17,19%	3	6,67%
Short of breath in the past 12 months	18	(16.8)	11	17,19%	7	15,56%
Difficulty of walking for another reason than heart or lung problems	14	(13.0)	10	15,63%	4	8,89%
Wheezing in the past 12 months in the past 12 months	12	(11.0)	9	14,06%	3	6,67%
Short of breath at rest last 12 months	6	(5.6)	4	6,25%	2	4,44%
Short of breath after intense effort last 12 months	20	(18.4)	13	20,31%	7	15,56%
Woken by an attack of shortness of breath in the past 12 months	3	(2.8)	3	4,69%	0	0,00%
Asthma	10	(9.3)	6	9,38%	4	8,89%
Nasal allergy	9	(8.3)	7	10,94%	2	4,44%
Respiratory distress when handling plants reaped	48	(44.0)	29	45,31%	19	42,22%
Severe respiratory problems interfered with usual daily activities	7	(6.4)	5	7,81%	2	4,44%
Chronic illness*	19	(39.6)	14	21,88%	5	11,11%
Early Airways Obstruction*	14	(29.8)	9	14,06%	5	11,11%

*n = 48; Early Airways Obstruction (FEF25–75 < 80% of the predicted level and FEV / FVC > 70%, after bronchodilation during the reversibility test)

### Description of air quality in the farms (dwellings, stables and granaries)

We present the characteristics, distributions of air pollutants in the farms and the relationships between respiratory outcomes and elevated exposure to air pollutants in the whole farm, and inside the dwellings and in granary/stable respectively.

#### VOC distribution

Table 3 shows the proportion of farms with a VOC concentration below the limit of detection (LOD) and between the LOD and the limit of quantification (LOQ) in living room, granary/stable respectively. Five VOCs were found under the LOD in more than 50% of the living rooms, and four VOCs had 100% of the value of the upper LOQ. In more than 50% of the stables, 10 VOCs were present below the LOD and 3 VOCs had 100% of the value of the upper LOQ. In more than 50% of the granaries, 7 VOCs were detected under the LOD, and 4 VOCs had 100% of the value of the upper LOQ.

**Table 3:** Proportion of farms (%) with Volatile Organic Compounds (VOCs) concentrations below the limit of detection (LOD) or between the LOD and the limit of quantification (LOQ) in the farm (living room, stable and granary respectively)

VOCs (μg/m^3^)	LOD (μg/m^3^)	LOQ (μg/m^3^)	% <LOD	% [LOD, LOQ]	% <LOD	% [LOD, LOQ]	% <LOD	% [LOD, LOQ]
			Living Room		Stable		Granary	
Acetaldehyde	0.3	0.4	0.0	0.0	0.0	3.0	0.0	0.0
Acrolein	0.1	0.3	33.3	0.0	93.9	0.0	95.5	0.0
Formaldehyde	0.6	1.1	0.0	3.9	24.2	42.4	18.2	31.8
Benzene	0.4	1.1	0.0	42.0	0.0	39.4	0.0	36.8
1,4-Dichlorobenzene	0.07	0.2	27.3	22.0	90.9	6.1	79.0	15.8
Etylbenzene	0.3	0.9	9.1	23.6	51.5	33.3	15.8	47.4
n-Decane	0.07	0.2	0.0	0.0	0.0	0.0	0.0	0.0
n-Undecane	0.5	1.4	0.0	0.0	0.0	0.0	0.0	0.0
Styrene	0.1	0.3	0.0	7.3	0.0	51.5	0.0	31.6
Tetrachloroethylene	0.4	1.2	98.2	1.8	100	0.0	100	0.0
Toluene	0.4	1.3	0.0	7.3	0.0	48.5	0.0	21.5
Trichloroethylene	0.4	1.0	94.6	1.8	97.0	3.0	94.7	0.0
1, 2,4-Trimethylbenzene	0.03	0.1	0.0	0.0	0.0	0.0	0.0	0.0
m/p-Xylene	0.5	1.5	10.9	14.6	54.6	24.2	15.8	31.6
o-Xylene	0.2	0.6	6.0	20.0	33.3	48.5	15.8	31.6
2-Butoxyethanol	0.4	1.5	43.6	38.2	87.9	6.1	84.2	15.8
2-Butoxyetylacetate	0.3	1.0	80.0	18.2	84.9	15.1	100	0.0
1-Metoxy-2-Propanol	0.5	1.8	52.7	34.6	97.0	3.0	84.2	5.3
1-Metoxy-2-propylacetate	0.7	2.2	90.9	9.1	100	0.0	89.5	10.5

*LOD* limit of detection is the lowest quantity of a substance that can be distinguished from the absence of that substance (a blank value) with a stated confidence level (generally 99%)

*LOQ* limit at which the difference between two distinct values of a substance can be reasonably discerned

All farms had VOCs measured in the living room. However, only 33 farms had VOCs measurements done in the stable, and 19 farms had measurements done in the granary. The distribution of VOCs in the living room, stable and granary is presented in the Table 4. Most VOCs had a higher mean in the living room, followed by the granary. The stables generally had the lowest VOC means. The total VOCs score ranged between 0 and 13 out of a possible 19 (median: 5 and 3rd quartile: 7). Correlation coefficients between VOCs ranged between 0.01 (between acetaldehyde and 1,4-Dichlorobenzene) and 0.99 (between o-Xylene and m/p-Xylene) (results not shown).

**Table 4:** Distribution of Volatile Organic Compounds (VOCs) in the farms

VOCs^a^	Place	Mean (μg/m^3^)	Min (μg/m^3^)	Median (μg/m^3^)	3rd quartile (μg/m^3^)	Max (μg/m^3^)
Acetaldehyde	L	18.60	1.53	16.64	23.76	66.70
	S	10.83	0.33	5.86	14.47	62.85
	G	14.95	1.26	8.97	18.97	74.28
Acrolein	L	0.66	0.00	0.51	0.77	3.80
	S	0.07	0.00	0.00	0.00	1.63
	G	0.08	0.00	0.00	0.00	1.26
Formaldehyde	L	15.01	0.39	13.04	19.32	59.52
	S	2.53	0.25	0.83	1.24	22.16
	G	3.04	0.26	1.18	2.27	24.15
Benzene	L	1.54	0.52	1.17	1.92	4.86
	S	1.29	0.62	1.12	1.52	2.66
	G	1.72	0.77	1.17	1.62	10.56
1,4-Dichlorobenzene	L	2.80	0.00	0.19	3.16	43.94
	S	0.09	0.00	0.03	0.06	1.26
	G	0.13	0.00	0.06	0.13	1.64
Etylbenzene	L	3.76	0.14	1.55	5.12	25.11
	S	0.77	0.14	0.25	0.43	6.47
	G	1.34	0.14	0.74	1.82	5.66
n-Decane	L	35.39	2.26	14.66	38.26	252.89
	S	10.59	2.20	5.03	6.84	96.93
	G	10.34	2.95	8.99	12.41	30.73
n-Undecane	L	42.35	3.51	24.34	47.03	248.53
	S	14.58	1.66	8.02	10.80	132.33
	G	12.47	1.89	9.88	16.59	33.60
Styrene	L	0.90	0.14	0.70	1.11	3.93
	S	0.39	0.14	0.29	0.39	1.32
	G	0.72	0.14	0.55	0.91	2.29
Tetrachloroethylene	L	0.08	0.00	0.05	0.05	0.55
	S	0.07	0.05	0.05	0.05	0.16
	G	0.09	0.05	0.05	0.11	0.22
Air Pollutant	Place	Mean	Min	Median	3rd quartile	Max
Toluene	L	12.09	0.45	6.10	13.41	105.82
	S	2.13	0.45	1.30	2.16	11.19
	G	5.89	0.54	3.23	5.26	28.64
Trichloroethylene	L	0.10	0.00	0.00	0.00	2.05
	S	0.03	0.00	0.00	0.00	0.72
	G	0.28	0.00	0.00	0.00	3.23
1, 2,4-Trimethylbenzene	L	25.16	0.89	8.43	20.04	172.69
	S	4.78	0.57	1.24	2.25	58.73
	G	6.88	0.76	4.19	9.07	24.10
m/p-Xylene	L	11.24	0.26	3.76	13.94	81.82
	S	2.24	0.16	0.47	0.89	29.19
	G	3.69	0.16	1.83	4.96	17.34
o-Xylene	L	4.82	0.17	1.69	5.31	30.77
	S	1.12	0.11	0.28	0.40	15.47
	G	1.53	0.11	0.79	2.09	6.44
2-Butoxyethanol	L	2.00	0.00	0.43	1.07	87.20
	S	0.27	0.00	0.00	0.07	3.94
	G	0.18	0.00	0.00	0.50	0.72
2-Butoxyetylacetate	L	0.14	0.00	0.00	0.27	1.08
	S	0.09	0.00	0.00	0.18	0.72
	G	0.00	0.00	0.00	0.00	0.00
1-Metoxy-2-Propanol	L	1.33	0.00	0.42	0.84	13.68
	S	0.05	0.00	0.00	0.00	0.68
	G	0.54	0.00	0.10	1.36	2.04
1-Metoxy-2-propylacetate	L	0.19	0.00	0.00	0.00	2.02
	S	0.02	0.00	0.00	0.00	0.59
	G	0.15	0.00	0.00	0.00	0.98
Global VOCs score			1	5	7	13

*Min* minimum, *Max* Maximum, *L* Living room, *S* Stable, *G* Granary

^a^For each VOC, 79 measurements were in the Living room for 50 farms 48 measurements in stables of 33 farms and 23 measurements in the granary of 19 farms

#### PM distribution

Table 5 shows the distribution of PM in the farms. The living room presented the highest mean UFPM number concentration (19298.0/cm^3^), with the highest median and the highest 3rd quartile (10478.0/cm^3^ and 19390/cm^3^, respectively). However, the maximum value of particle number concentration was observed in the bedroom (229300/cm^3^). In terms of mass concentration, granary/stable had the highest mean, median and quartile of PM_2.5_ (13.3 μg/m^3^, 2.5 μg/m^3^, 8 μg/m^3^, respectively), PM_10_ (259.6 μg/m^3^, 69.5 μg/m^3^, 167 μg/m^3^, respectively) and TSP (361.8 μg/m^3^, 156.5 μg/m^3^, 334 μg/m^3^, respectively). The living room and the bedroom had similar PM values.

**Table 5:** Distribution of particulate matter (PM) in the farms

Air Pollutants	Place	Mean	Min	Median	3rd quartile	Max
UFPM (pt/cm^3^)	L	19298.0	631	10478.0	19390	148466
	BR	17534.1	262	4928.0	18300	229300
	G/S	7066.8	725	3529.0	7385	41920
PM_1_ (μg/m^3^)	L	0.9	0	0.0	0	17
	BR	1.0	0	0.0	0	8
	G	0.8	0	0.0	1	12
PM_2.5_ (μg/m^3^)	L	4.0	1	2.0	4	40
	BR	4.5	0	1.0	3	46
	G/S	13.3	0	2.5	8	327
PM_10_ (μg/m^3^)	L	30.7	10	25.0	32	159
	BR	26.4	0	16.0	28	159
	G	259.6	1	69.5	167	5733
TSP (μg/m^3^)	L	48.4	13	37.0	55	208
	BR	46.3	0	26.0	42	452
	G/S	361.8	1	156.5	334	5561

*UFPM* Ultrafine particulate matter, *PM* Particulate Matter, *TSP* Total Suspended Particles, *L* Living room, *BR* Bed Room, *G/S* Granary/Stable

### Relationships of air pollution to respiratory health

#### Air pollution in the whole farm

When total exposure to air pollution was considered taking into account average exposure in both the dwellings and workplaces, there was a trend for an increased risk of asthma in case of elevated exposure to the halogenated hydrocarbons families as assessed through the score (adjusted odds-ratio (ORa) = 2.02, 95% CI: 1.00–4.10) and to benzene (ORa = 4.11, 95% CI: 0.91–18.5) (Table 6). The risk for early airway obstruction was significantly increased in the case of elevated exposure to 2-butoxyetylacetate (ORa = 11.49, 95% CI: 1.55–85.37) (Table 6). The risk of COPD was found to be larger with elevated exposure to aldehydes (OR = 3.95, 95% CI: 1.09–14.26),. No significant relationship was found between respiratory outcomes and the total VOCs score.

**Table 6:** Relationships between elevated exposure to VOCs and asthma and early airway obstruction in the farmers

VOCs	Asthma				Early Airways Obstruction			
	Crude OR (95% CI)		Adjusted OR (95% CI)		Crude OR (95% CI)**		Adjusted OR (95% CI)**	
Acetaldehyde	0.34	0.04–2.71	–	–	1.59	0.37–6.83	2.68	0.33–22.00
Acrolein	0.37	0.05–3.04	0.14	0.03–0.75	1.48	0.23–9.47	1.99	0.19–21.11
Formaldehyde	0.31	0.04–2.51	0.31	0.04–2.51	2.48	0.49–12.65	1.88	0.28–12.50
Benzene	2.45	0.67–8.96	4.11	0.91–18.5	2.08	0.41–10.60	1.37	0.19–9.72
1,4-Dichlorobenzene	3.00	0.91–9.93	5.62	0.83–37.91	0.68	0.12–3.86	0.98	0.15–6.45
Etylbenzene	0.75	0.15–3.72	0.40	0.08–1.90	0.85	0.15–4.95	1.01	0.14–7.19
n-Decane	1.06	0.27–4.17	1.03	0.25–4.17	0.63	0.11–3.58	1.87	0.18–19.40
n-Undecane	1.28	0.35–4.65	2.43	0.56–10.19	0.28	0.03–2.58	0.41	0.02–9.10
Styrene	0.33	0.041–2.69	–	–	3.56	0.81–15.66	8.86	0.65–119.99
Tetrachloroethylene	1.56	0.42–5.89	1.40	0.38–5.08	1.49	0.30–7.33	7.78	0.59–102.53
Toluene	0.57	0.12–2.86	0.37	0.07–1.97	1.40	0.29–6.85	2.43	0.42–14.14
Trichloroethylene	1.60	0.35–7.46	3.20	0.59–17.21	–	–	–	–
1, 2,4-Trimethylbenzene	0.34	0.04–2.78	0.41	0.09–1.91	0.82	0.14–4.81	1.08	0.09–12.36
m/p-Xylene	0.75	0.15–3.72	0.36	0.08–1.72	0.85	0.15–4.95	1.01	0.14–7.19
o-Xylene	0.75	0.15–3.72	–	–	0.85	0.15–4.95	1.01	0.14–7.19
2-Butoxyethanol	1.27	0.33–4.81	0.75	0.19–2.92	3.62	0.81–16.12	6.48	0.59–71.33
2-Butoxyetylacetate	0.53	0.11–2.48	0.26	0.06–1.26	**10.2**	**2.14–48.84**	**11.49**	**1.55–85.37**
1-Metoxy-2-Propanol	0.28	0.03–2.45	0.44	0.05–4.28	0.76	0.17–3.45	1.44	0.22–9.41
1-Metoxy-2-propylacetate	0.26	0.03–2.20	0.28	0.03–2.92	1.50	0.35–6.46	2.17	0.41–11.47
Halogenated hydrocarbons score	1.63	0.83–3.2	**2.02**	**1.00–4.10**	0.68	0.29–1.63	0.92	0.38–2.25
Glycolether score	0.69	0.39–1.22	0.60	0.33–1.12	1.58	0.94–2.66	1.86	0.93–3.69
Aromatic hydrocarbons	0.92	0.65–1.30	0.86	0.63–1.18	1.07	0.79–1.46	1.15	0.82–1.62
Aliphatic hydrocarbons	1.09	0.55–2.16	1.31	0.64–2.69	0.63	0.23–1.70	0.95	0.25–3.56
Aldehydes	0.45	0.11–1.37	0.23	0.06–0.78	1.63	0.73–3.68	1.69	0.59–4.80

Early Airways Obstruction: (FEF25–75 < 80% of the predicted level and FEV / FVC > 70%, after bronchodilation during the reversibility test),

OR: odds ratios between mean concentration in the farm (living room, stable, granary) and health outcomes; (95% CI): 95% confidence interval; Adjusted OR (ORa): odds ratio adjusted for gender, age, smoking habit, relative humidity, presence of pets and presence of mold;

Bold values in bold denote statistically significant estimates of OR (*p* < 0.05)

#### Air pollution in the dwellings

**Fig. 1:**
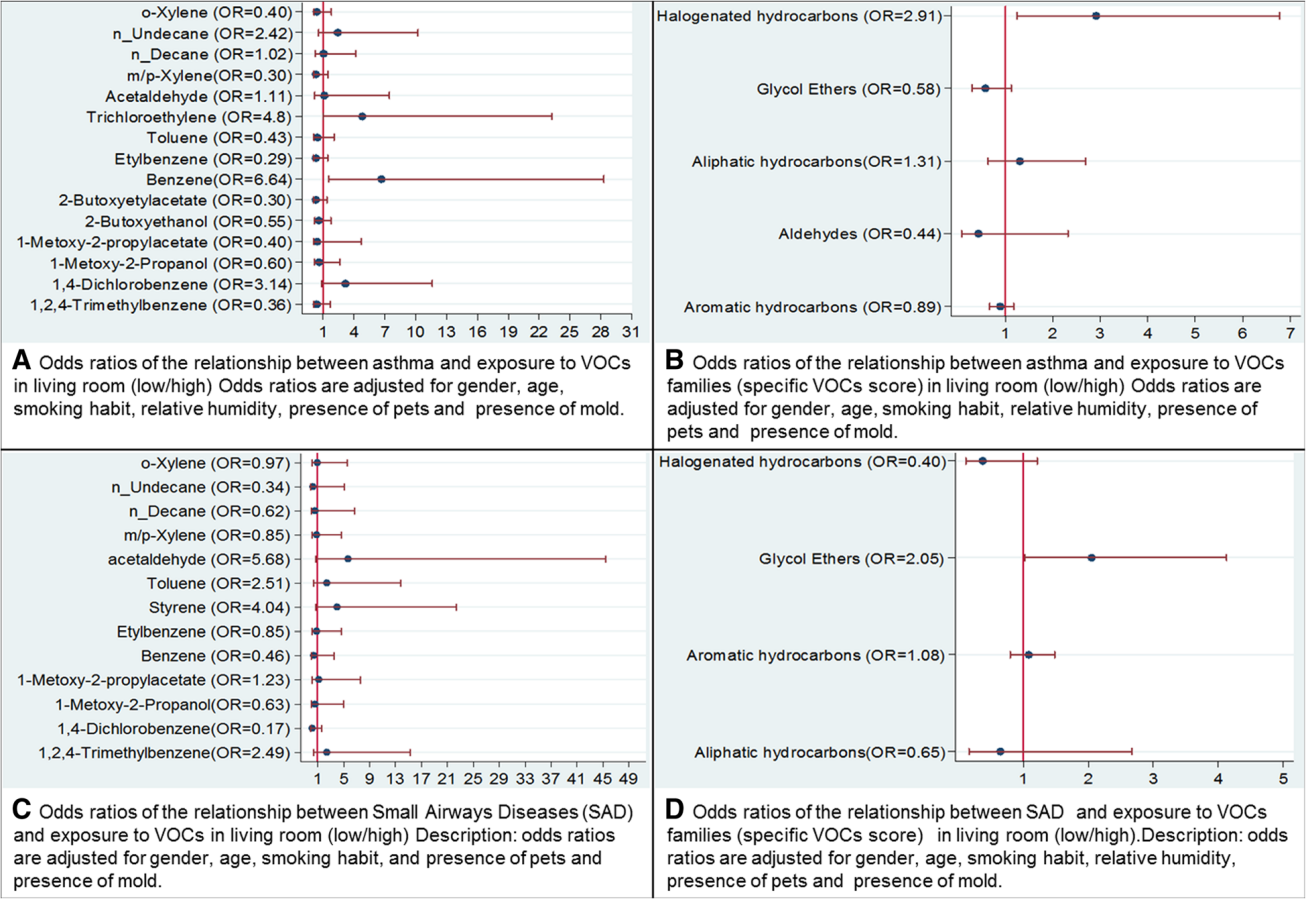
Adjusted odds ratios (OR) and confidence interval of the relationship between respiratory health and exposure to Volatile Organic Compounds (VOCs) in the living room

Benzene and trichloroethylene assessed in the living room were significantly associated with asthma for benzene (ORa = 6.64, 95% CI: 1.56–28.27) and for trichloroethylene (ORa = 4.8, 95% CI: 1.0–23.3) (Fig. 1a). No other significant relationships were found (Fig. 1a). Marginal models did not converge for tetrachloroethylene, which had concentrations below the LD in most dwellings, or acrolein and formaldehyde, which had many missing values. A trend was observed between acetaldehyde and styrene in the living room and early airway obstruction after adjusting for confounder variables (Fig. 1c). No association was found between PM or single VOC and COPD (data not shown). The total VOCs score was not related to asthma, COPD or early airway obstruction after adjusting for confounder variables (data not shown). However, the halogenated hydrocarbons score was significantly associated with an increasing occurrence of asthma (ORa = 2.9; 95% CI: 1.3–6.8) (Fig. 1b) and the glycol ethers score with an increasing occurrence of early airway obstruction (OR = 2.0; 95% CI: 1.0–4.1) (Fig. 1d).

#### Air pollution in the workplaces (granary/stable)

Exposure to VOCs in stables or granaries was not related to respiratory outcomes, as no association was found between the total and specific VOCs scores and the health outcomes. Being exposed to elevated fine particles < 2.5 μm (μg/m^3^) in the stable and granary was significantly related to early airway obstruction after adjusting for confounders (ORa = 5.26, 95% CI: 1.09–25.28) (Table 7).

**Table 7:** Relationships between elevated exposure to particulate matter (PM) and asthma and Early Airways Obstruction in the stable and granary in the farmers

PM	Asthma				Early Airways Obstruction			
	cOR^a^ (95% CI)^b^		aOR^†^ (95% CI)		cOR (95% CI)		aOR^†^ (95% CI)	
Average Particles number (pt/cm^3^)	2.48	0.68–8.97	2.43	0.50–11.80	1.65	(0.33–8.39)	1.85	(0.32–10.59)
PM_1_ (μg/m^3^)	0.63	0.14–2.91	1.07	0.19–5.84	0.56	(0.13–2.52)	0.50	(0.08–3.04)
PM_2.5_ (μg/m^3^)	0.29	0.04–2.42	0.30	0.05–1.69	**5.31**	**(1.25–22.65)**	**5.26**	**(1.09–25.28)**
PM_10_ (μg/m^3^)	1.28	0.34–4.79	2.20	0.38–12.55	1.32	(0.31–5.58)	1.10	(0.22–5.42)
TSP (μg/m^3^)	0.88	0.22–3.54	1.59	0.27–9.21	1.67	(0.39–7.13)	0.97	(0.15–6.44)

Early Airways Obstruction: (FEF25–75 < 80% of the predicted level and FEV / FVC > 70%, after bronchodilation during the reversibility test)

*UFPM* Ultrafine particulate matter, *PM* Particulate Matter, *TSP* Total Suspended Particles

^a^*OR* odds ratios

^b^(95% CI): 95% confidence interval

^†^aOR: Adjusted odds ratio adjusted for gender, age, smoking habit, relative humidity, presence of pets and presence of mold: values in bold denote statistically significant estimates of OR (*p* < 0.05)

## Discussion

### Main findings

This study explored the relationship between respiratory health and exposure to PM and VOCs in French farmers in the Auvergne region. Asthma was found to be significantly associated with both benzene and trichloroethylene and early airway obstruction with PM_2.5_. When taking the specific VOCs families into account, asthma was positively associated with the halogenated hydrocarbons and early airway obstruction with glycol ethers. In addition, the risk of COPD was found to be larger with elevated exposure to aldehydes but this condition was rare in our population. To our knowledge, few studies have assessed chemical air pollution in farms and related it to farmers’ respiratory health, which is important in understanding the development and the aggravation of chronic respiratory diseases in the rural settings.

### Literature

Our results are consistent with previous findings relating benzene to wheezing and asthma [15–17]. Few studies have assessed the impact of VOCs in rural areas In a previous study conducted in a rural area [18], we found that indoor benzene exposure, as indicated by *urinary* S-phenylmercapturic acid (*SPMA*), was significantly associated with asthma in children. The odds-ratio for asthma was eight times higher in highly exposed children compared with weakly exposed children (exposure superior to the median, OR = 8.11; CI 95%: 1.41–46.34). However, the association between benzene and asthma was significant only among non-atopic children (OR = 8.58; 95% CI: 1.4–51.59 vs. OR = 7.54; 95% CI: 0.55–103.21). These findings are important as infants and young children are particularly susceptible to developing respiratory diseases and the prevalence of asthma is still increasing among them [19–22].

Association between exposure to trichloroethylene and asthma is consistent with that observed in the literature. Trichloroethylene, in addition to being carcinogenic, has been found to induce respiratory symptoms such as rhinitis [10] and asthma [23]. Blair et al. [24] found that men exposed to trichloroethylene from maintenance work on aircraft for at least 1 year between 1952 and 1956 in the U.S. state of Utah died more often from asthma than men in the general Utah population. Using a specific score for halogenated hydrocarbons (trichloroethylene, 1,4-dichlorobenzene and tetrachloroethylene), we also found a significant increase in asthma prevalence. Among the three compounds in this group, only trichloroethylene had a significant link with asthma. When considering the outdoor exposure in the farm, 2-butoxyetylacetate was found positively associated with early airway obstruction. To our knowledge, these are novel findings. In addition, the analysis of the specific VOC score indicated that glycol ethers were significantly associated with early airway obstruction, which suggests that either a combined action of pollutants contributes to early airway obstruction or they are proxy of exposure to other pollutants.

The mechanisms by which VOCs may act on respiratory health are based on irritation [25] or on the alteration of cytokine levels that may be involved in the inception of respiratory diseases, such as COPD in farmers due to their exposure to these chemicals [26].

These findings are consistent with previous studies which focused on the effect of multi-pollution [9, 10]. In the adult population, exposure to aromatic compounds has been found to be significantly associated with physician-diagnosed asthma (adjusted OR = 1.63, 95% CI: 1.17–2.27) [9]. In addition, total VOCs has been found associated with an increasing risk of shortness of breath in adults [26] and asthma in children [16].

The association between PM_2.5_ and early airway obstruction is consistent with the results observed by Chrug et al. [27] that examined samples taken from the lungs of 20 women from Mexico City, a high PM zone, and control samples from 20 never-smoking, non-dust-exposed subjects from Vancouver, British Columbia, Canada, a low PM region. Women from Mexico City had abnormal small airways associated with fibrotic walls and excess muscle, of which many contained visible dust. The conclusion was that PM was retained in the walls of small airways and that, even in non-smokers, long-term exposure to high levels of ambient particulate pollutants was associated with early airway obstruction.

We used the 3rd quartile value in this investigation in order to categorize VOCs on the farms, and we defined a high exposure in the case of a concentration higher than this cut-off point. This quartile was used to create a total score of exposure to VOCs in a previous study where significant associations between total score VOCs and asthma and rhinitis (odds ratio (OR) of 1.40 and 1.22, respectively) were found [10]. In our study, the association between the total VOCs score and asthma was not significant. This result could be explained by the higher level of exposure observed by Billionnet et al. [10] as compared to our measures in a rural area. For example, the median and maximum values of benzene in the study by Billionnet et al. were 13 μg/m^3^ and 368.5 μg/m^3^, respectively; whereas in our case, the median and maximum values of benzene in the living room were 1.2 μg/m^3^ and 4.9 μg/m^3^, respectively. The maximum value of benzene we found in the farm living room was even lower than the limit value of benzene in the atmosphere, according to the 2010 regulations of the European Commission (5 μg/m^3^). Therefore, the exposure levels of VOCs that we measured on the rural farms are lower than those in urban areas. This finding is consistent with the observation by Hulin et al. [25] that urban dwellings are more polluted than rural ones, with concentrations up to two times higher. As a whole, our results support the hypothesis that even low level of air pollutants may have adverse health effects.

### Study strengths and limitations

While there have been studies employing a multi-pollutant approach to assess exposure to several VOCs in urban dwellings [9, 10, 16], this study contributes to the literature by bringing a multi-pollutant analysis to a rural area where potential sources of VOCS, both indoor and outdoor, are numerous.

This study presents certain limitations. Apart from early airway obstruction and COPD, which were objectively assessed through spirometry with each curve checked by an author, health outcomes of our study were obtained using a standardized self-administered questionnaire. This kind of data collection may have inherent bias for all the declarative data [28, 29]. In addition, the number of farms participating in the study was limited, and the investigation was conducted only in the Auvergne region, which is not representative of other farming regions of France. In terms of smoking, we do not know whether our farmers were representative in terms of smoking habit. Indeed, a recent study showed that active smoking prevalence was similar in farmers and in non-farmers [30]. Due to the low frequency of COPD in the data from pulmonary function and gold criteria, marginal models did not converge for most VOCs and there were missing values. Thus, we were unable to perform analyses using COPD data. An additional value results from the fact that we took into account early airway obstruction, which is original. However, the definition of early airway obstruction we used is not standardized and results obtained have to be taken cautiously.

Due to the cross-sectional design of the study, temporal relationships between VOCs and PM exposure and asthma cannot be established with certainty and does not allow for assessing causality. Measurements were conducted over the course of a few weeks, and may be a poor surrogate for past year exposure. Lastly, although indoor and outdoor exposures were measured, we cannot rule out the possibility that respiratory symptoms were due to past indoor or outdoor exposures.

## Conclusions

In conclusion, our findings, drawn from a population-based sample, suggest that VOCs and PM may be linked with respiratory health, namely asthma and early airway obstruction, in farmers. This cross-sectional study lays the foundation for further cohort or toxicological studies in order to confirm these associations and test for causality.

## Data Availability

Data are available upon request.

## Abbreviations

COPD: Chronic Obstructive Pulmonary Diseases
DNPH: Dinitrophenylhydrazine
FERMA: Environmental Factors of Allergic and Respiratory Diseases study
FEV_1_: Forced expiratory volume
FVC: Forced vital capacity
GEE: Generalized estimating eq.
LD: Limit of detection
MSA: “Mutuelle des Salariés Agricoles”
ORa: Adjusted odds-ratio
PM: Particulate matter
SD: Standard deviation
TSP: Total suspended particles
VOCs: Volatile organic compounds
